# A pan-cancer analysis uncovering the function of CRHBP in tumor immunity, prognosis and drug response: especially its function in LIHC

**DOI:** 10.1038/s41598-024-52387-8

**Published:** 2024-02-07

**Authors:** Bangjie Chen, Sanwei Chen, Xinyi Wang, Jianlin Zhang, Hanying Wang, Jiajin Li, Ziyu Zhang, Feng Yu, Weihao Kong

**Affiliations:** 1 Department of Oncology, the First Affiliated Hospital of Anhui Medical University, Hefei, China; 2 Department of General Surgery, the Second Affiliated Hospital of Anhui Medical University, Hefei, China; 3 Department of Radiation Oncology, the First Affiliated Hospital of Anhui Medical University, Hefei, China; 4 Department of Emergency Surgery, the First Affiliated Hospital of Anhui Medical University, Hefei, China; 5 Department of Operation Theater, First Affiliated Hospital of Anhui Medical University, Hefei, China; 6 The Second Clinical Medical College of Anhui Medical University, Hefei, China; 7 The First Clinical Medical College of Anhui Medical University, Hefei, China; 8 Department of Emergency Medicine, The First Affiliated Hospital of Anhui Medical University, Hefei, China; 9 Gastrointestinal Surgery Department, West China Hospital of Sichuan University, Chengdu, China

**Keywords:** Cancer, Tumour biomarkers

## Abstract

Corticotropin-releasing hormone-binding protein (CRHBP) is involved in many physiological processes. However, it is still unclear what role CRHBP has in tumor immunity and prognosis prediction. Using databases such as the Cancer Genome Atlas (TCGA), Gene Expression Omnibus (GEO), Tumor Protein Database, Timer Database, and Gene Expression Profiling Interactive Analysis (GEPIA), we evaluated the potential role of CRHBP in diverse cancers. Further research looked into the relationships between CRHBP and tumor survival prognosis, immune infiltration, immune checkpoint (ICP) indicators, tumor mutation burden (TMB), microsatellite instability (MSI), mismatch repair (MMR), DNA methylation, tumor microenvironment (TME), and drug responsiveness. The anticancer effect of CRHBP in liver hepatocellular carcinoma (LIHC) was shown by Western blotting, EdU staining, JC-1 staining, transwell test, and wound healing assays. CRHBP expression is significantly low in the majority of tumor types and is associated with survival prognosis, ICP markers, TMB, and microsatellite instability (MSI). The expression of CRHBP was found to be substantially related to the quantity of six immune cell types, as well as the interstitial and immunological scores, showing that CRHBP has a substantial impact in the TME. We also noticed a link between the IC50 of a number of anticancer medicines and the degree of CRHBP expression. CRHBP-related signaling pathways were discovered using functional enrichment. Cox regression analysis showed that CRHBP expression was an independent prognostic factor for LIHC. CRHBP has a tumor suppressor function in LIHC, according to cell and molecular biology trials. CRHBP has a significant impact on tumor immunity, treatment, and prognosis, and has the potential as a cancer treatment target and prognostic indicator.

## Introduction

CRHBP, a 37 kDa secreted glycoprotein, was found in human plasma as a molecule that interacted with corticotropin-releasing hormone (CRH) radioimmunoassays^1^. In all vertebrates, CRHBP possesses a highly folded tertiary structure that maintains 10 cysteine residues in 5 consecutive disulfide linkages^2,3^. As a member of the CRH family, CRHBP differs structurally from CRHR (CRH receptor) and has higher affinity for binding CRH and UCN1 (urocortin I) than CRHR, but variable or less affinity for UCN2 (urocortin II) and UCN3 (urocortin III)^4,5^. The CRH and CRHR physiological responses are modulated by CRHBP, which is associated with the hypothalamic–pituitary–adrenal (HPA) axis^2,6,7^. It can control a range of physiological responses, including immunological responses^8^, metabolism^9^, and stress responses^1,10–14^. It also decreases the release of CRH-induced adrenocorticotropic hormone (ACTH).

Multiple diverse tissues and organs, such as the liver, kidney, brain, and placenta, express CRHBP^3,13^. Recent research has linked it to the development of several cancers^6,10,15^. Liver hepatocellular carcinoma (LIHC) tissues have significantly lower levels of CRHBP expression than normal liver tissues, according to research by Xia et al., CRHBP expression was also inversely connected with survival^16^. In contrast, Yang et al. discovered that CRHBP overexpression inhibited the growth of renal cell carcinoma (RCC), reducing numerous functions such as proliferation and invasion. Furthermore, they discovered that CRHBP might alter inflammation and apoptosis in clear renal cell carcinoma (ccRCC) via a putative mechanism targeting NF-B and p53-mediated mitochondrial apoptotic pathways^6^. Moreover, it has been demonstrated that a higher risk of breast cancer is linked to lower CRHBP expression in Caucasians and Black Americans (BC)^17^. Similar evidence of reduced CRHBP mRNA and protein expression was discovered in investigations relating to bladder cell carcinoma and prostate cancer^10^. Wang et al. revealed that CRHBP is related to ovarian plasmacytoid cystic adenocarcinoma (OVSC) survival and prognosis, and they also found that CRHBP may be engaged in a number of functions, including angiogenesis, hormone receptor binding, and T cell activation through functional enrichment analysis^18^.

These studies have revealed the role of CRHBP in many tumor species, suggesting that CRHBP may be a potential biomarker for immunotherapy and prognosis prediction. At present, oncologists are keen to find therapeutic targets for pan-tumor, including targeted therapy and immunotherapy. For example, the recently discussed AOH1996 is an anti-tumor drug targeting PCNA. City of Hope National Medical Center, the top cancer research institution in the United States, reported that in preclinical studies, AOH1996 can kill almost all solid tumors. In this context, based on the special significance of CRHBP in various tumors, it is necessary for us to conduct a pan-cancer analysis, which is the first time. In addition, the role of CRHBP in human immunotherapy and prognosis has not been well studied, and further research is needed to determine the mechanism by which it affects the malignant biological activity of tumors. Notably, we identified CRHBP as an independent predictor of LIHC prognosis through univariate and multivariate regression analyses. Therefore, we also used cellular and molecular biological methods to explore the impact of CRHBP on the basic tumor phenotype and function of LIHC.

## Material and methods

### Data and software availability

The Cancer Genome Atlas (TCGA) (https://cancergenome.nih.gov/) and the Gene Expression Omnibus (GEO) databases (https://www.ncbi.nlm.nih.gov/geo/) were used to retrieve all raw data sets. R3.2.3 is used for integration processing of raw data and validation of database analysis results. All our applied online web tools are described individually in the following paragraphs.

### CRHBP expression analysis in normal and malignant tissues

Using the UCSC cancer genome browser (https://tcga.xenahubs.net), we retrieved TCGA level 3 RNA sequencing processing data and associated clinical annotations for 33 cancers. Due to the TCGA database's very low data on normal tissue RNA sequences, we collected transcriptome data from the Genotype Tissue Expression database (GTEx; https://www.gtexportal.org/) to evaluate the levels of CRHBP expression in tumour and normal samples. Additionally, the Oncomine database (https://www.oncomine.org/resource/login.html), TIMER (https://cistrome.shinyapps.io/timer/), GEPIA database (http://gepia2.cancer-pku.cn/#analysis), and CCLE database (https://portals.broadinstitute.org/ccle/) were also used to detect CRHBP expression between malignancy and matched normal tissue (NS means p ≥ 0.05; * means p < 0.05; ** means p < 0.01; *** means p < 0.001).

Furthermore, we examined the expression levels of CRHBP in the major pathological stages (I, II, III, IV, V) of breast invasive carcinoma (BRCA), renal and renal clear cell carcinoma (KIRC), lung adenocarcinoma (LUAD), pancreatic cancer (PAAD), testis germinoma (TGCT), and thyroid carcinoma (THCA) by means of TCGA data.

### Study of CRHBP's predictive significance in cancer

Using the GEPIA (http://gepia.cancer-pku.cn/) and prognoscan datasets (http://dna00.bio.kyutech.ac.jp/PrognoScan/index.html), we explored the prognostic importance of CRHBP for survival in human malignancies. Data on tumor and normal tissue may be evaluated using the online GEPIA database, which is created from the TCGA database. We produced survival maps of CRHBP expression across TCGA tumors using the "survival analysis" module of GEPIA2 and explored the connection between CRHBP expression and overall survival (OS) and disease-free survival (DFS) in 33 different cancer types. The Kaplan–Meier Plotter database (http://kmplot.com/analysis/) was used to automatically identify the groups using the optimal cutoff values. When the log-rank test was employed to test a hypothesis, the appropriate 95% confidence intervals (CI) and risk ratios (HRs) for log-rank P values were established.

### Analysis of the relationship between CRHBP expression, immunological infiltration, and immune checkpoint markers

The first step we took was utilize SangerBox website to examine the correlation between the levels of CRHBP expression and the numbers of six immune cells found in the tumor micro-environment (TME): B cells, CD4 + T cells, CD8 + T cells, neutrophils, macrophages, and dendritic cells. We revisited the relation between CRHBP expression and tumor-infiltrating lymphocytes (TILs) using the TIMER databases (https://cistrome.shinyapps.io/timer/) and GEPIA datasets in order that we could confirm the earlier results.

Correlations between ImmuneScore, StromalScore and ESTIMATEScore and CRHBP expression are subsequently presented in the form of the top 3 cancers, respectively. Higher ImmuneScore or StromalScore scores indicate a higher percentage of the associated component content and are positively connected with the proportion of immunity or stroma in the TME, respectively, while the ESTIMATEScore indicates the mixture of the two components in the TME. Using the "ESTIMATES" package for R, we were able to identify the ImmuneScore and the StromalScore.

Using the TIMER database, we also checked the We also examined the association between CRHBP expression levels and essential immunological checkpoint markers, such as ADORA2A, BLTA, CTLA4, CD28, CD200R1, CD200, TNFSF4, CD244, LAG3, NRP1, LAIR1, CD40LG, HAVCR2, ICOS, ICOS, TNFRSF14, and CD276. Spearman correlation analysis was used to demonstrate the statistical significance of the P value of 0.05.

Last but not least, we examined human pan-cancer somatic cell data (MAF data) from the TCGA database using the "maftools" application in R software to evaluate the connection between CRHBP and TMB or MSI. Additionally examined was the amount of exon mutations that indicate TMB in each malignancy. The TCGA database's MSI scores were used. Using Spearman's correlation analysis, the linkage between CRHBP expression and TMB or MSI was investigated.

### Examination of CRHBP expression in several immunological and molecular subtypes of pan-cancer

We studied the expression of CRHBP in several immunological or molecular subtypes of various cancer types by using the TISIDB database (http://cis.hku.hk/TISIDB/index.php). An extensive pan-cancer dataset is made available through the TISIDB database, an online integrated repository site, from the TCGA database. Differences that were statistically significant were taken into consideration, when the P value < 0.05.

### An examination of the linkage between CRHBP expression, mismatch repair gene mutation, and DNA methylation

We applied the TCGA database to examine the link between the expression levels of five mismatch repair (MMR) genes (MLH1, MSH2, MSH6, EPCAM, and PMS2) and CRHBP in different cancers. We also evaluated the connections between the expression levels of four methyltransferases, including DNMT1, DNMT2, DNMT3A, and DNMT3B, and CRHBP expression via the identical methods.

### Analysis of the correlation between CRHBP expression and drug response

Using CellMiner (http://discover.nci.nih.gov/cellminer/), we hypothesized the relationship between CRHBP expression and drug response.

### Analysis of gene-related enrichment

In order to better understand the biological functions and pathways related to CRHBP, we performed gene set enrichment analysis (GSEA) on chemical and genetic disturbances gathered from the molecular characteristic database (MSigDB) H (marker gene set), as well as KEGG subsets of typical pathways and cancer modules. They were put into action on Sangerbox (http://sangerbox.com/). GSEA findings are shown using normalized enrichment scores (NES), which take into consideration the size and amount of overrepresentation of a gene set at the gene ranking list's top or bottom (p-value as 0.05 and FDR as 0.25). In addition, we complementarily used the Genemania (http://genemania.org/) website to construct a protein interaction network for CRHBP. Based on the protein interaction network, we input the interacting genes into the GSCA website to evaluate the relationship between the GSVA score and the activity of different tumor-related pathways. Lastly, we visualized the resulting enrichment maps using R software (http://www.r-project.org) and Bioconductor.

### Patient and tissue collection

Clinical Medical Research Ethics Committee of the First Affiliated Hospital of Anhui Medical University authorized the project (Quick-PJ 2023-05-30). The liver tissues were given by the General Surgery Division of Anhui Medical University's First Affiliated Hospital. According to WHO guidelines, all patients were diagnosed with LIHC and underwent drastic surgery. The surgical specimen is immediately immersed in liquid nitrogen for fast freezing before being stored in − 80 degrees refrigerator. All participating patients gave their informed consents. All research was performed in accordance with relevant guidelines. Research involving human research participants have been performed in accordance with the Declaration of Helsinki.

### Cell culture

After being collected from the Center for Excellence in Molecular Cell Science, the LIHC cell lines were then frozen in liquid nitrogen for later use. All of the cell lines were grown in DMEM Medium (Gibco BRL, USA), which was augmented with 10% fetal bovine serum (FBS), 100 U/ml penicillin, and 100 mg/ml streptomycin, and the temperature was maintained at 37 °C with 5% carbon dioxide.

### Plasmid transfection

Overexpressed plasmids of CRHBP have been created at our lab in the past, and they have been preserved there (PEX-3-CRHBP, Hefei, China). The PEX-3 vector was used as our unfavorable control for this experiment. In order to transfect PEX-3-CRHBP and PEX-3 into human LIHC cells, we make use of the liposome anticorrosive amine TM2000, which is suggested by the manufacturer.

### Western blotting

To separate total proteins, we adopt the radioimmunoprecipitation assay buffer reagent (RIPA; Beyotime, China) in conjunction with phenylmethanesulfonyl fluoride (PMSF; Sigma, USA). This is done in accordance with the guidelines provided by the manufacturer. The bicinchoninic acid (BCA) protein assay kit was used in order to assess the quantities of proteins (Beyotime, China). After electrophoresis on a 10% sodium dodecyl sulfate–polyacrylamide gel, the proteins that were successfully isolated from each sample were transferred to a polyvinylidene fluoride (PVDF) membrane (Millipore, USA). After that, phosphate-buffered saline (PBS) with 5% nonfat dry milk is utilized to block the cell membrane for an additional hour. Before being rinsed with TBST (TBS + Tween) for a total of 15 min, the transferred membranes were first subjected to an overnight incubation at 4 °C with the primary antibodies. This step was followed by three separate washes.

Secondary antibodies were then applied to the transferred membranes for a further hour at room temperature. Following carefully washing in TBST, examine the immunoblot using a Thermo Scientific chemiluminescence development kit and perform analysis with Bio-Quantity Rad's one software. Primary antibodies including anti-CRHBP, anti-MMP2, anti-MMP9, anti-Bax, anti-Bcl-2 and anti-PCNA were all Rabbit anti human antibodies, which were purchased from Abca, UK, and the dilution concentration was 1:1000 in the experiment. The secondary antibody was horseradish peroxidase labeled Rabbit anti human IgG antibody, which was purchased from Abca, UK, and the dilution concentration was 1:1000 in the experiment.

### Transwell assay

We employ transwell assays to assess cell migration. Human HepG2 cells and matrigel were infected in 100 ml of serum-free medium before being transferred to the top chamber. 600 cc of DMEM medium with 20% FBS filled the bottom chamber. Once the 24-well plate was removed from the 5% (v/v) CO_2_ incubator, the cells in the top chamber were fixed with 0.1% crystalviolet staining and 10% neutral buffered formalin solution for 15 and 20 min, respectively. Using the microscope, count cells in five random visual regions.

### Wound healing assay

A cell density of 1 × 10^6^ /ml was used to seed cells in logarithmic growth phase onto 6-well plates. Placing the plate in the incubator for 24 h until the cells reach 70% of the bottom area. Creating linear wounds and replacing the culture medium to FBS-free. The plates were placed in an incubator and allowed to grow for 24 h. Ten percent neutral buffered formalin solution was used to fix the cells for 20 min, and then 0.1% crystal violet solution was used to stain the cells for 15 min. Photographs were taken under an inverted microscope of linear wounds.

### EdU assay

A DNA in vitro kit for the EdU Apollo strain purchased from RIBOBIO in Guangzhou, China, was used for the analysis. To get cells to the stationary phase of development, they are thawed and resuspended while still in the logarithmic growth phase, then around 6 × 10^3^ cells are sown per well in a 24-well plate. Incubate the cells for 2 h at 37 °C/5% CO_2_ after an overnight culture with 50 μM EdU in the media. then paraformaldehyde 4%, glycine, and Triton X-100 1% should be used to treat the cells. The cells were then stained for 30 min with Hoechst and Apollo. Fluorescence microscopy was used to get the pictures (Olympus, Tokyo, Japan).

### JC-1 assay

Using JC-1 staining kit (Beyotime, China) to detect the difference of mitochondrial membrane potential (MMP) in different groups. Cells were washed with PBS one time, then added 1.5 ml JC-1 staining fluid and incubate it in the dark for 20 min. Subsequently, using 1 × cold staining buffer to wash the cells for twice. Finally, the MMPs of different samples were observed by fluorescence microscope (Olympus, Tokyo, Japan).

### Statistical analysis

The relation between CRHBP expression and the values of interest (immune cell infiltration score, TMB, MSI, MMR, and methyltransferase genes) was analyzed using the Spearman test for bioinformatics validation. Both the paired *t*-test and the unpaired *t*-test were used to compare the levels of CRHBP expression in pan-carcinoma tissues and normal tissues. Univariate and multivariate Cox regression analysis was used to analyze the independent prognostic value of clinical characteristics and CRHBP expression levels. A significance level of 0.05 was used to determine statistical significance. All graphs were created using ggplot2 and forestplot.

As for molecular biology validation, Statistical analysis was performed using SPSS (Statistical Product and Service Solutions). To determine if the differences between the two groups were statistically significant, a two-tailed student t-test was performed. The data are shown as a mean ± SD. A significance level of 0.05 was used to determine statistical significance.

### Ethics approval and consent to participate

Clinical Medical Research Ethics Committee of the First Affiliated Hospital of Anhui Medical University authorized the project (Quick-PJ 2023-05-30). All participating patients were informed and consented.

## Results

### The expression of CRHBP varies between cancerous and healthy tissues

**Figure 1:**
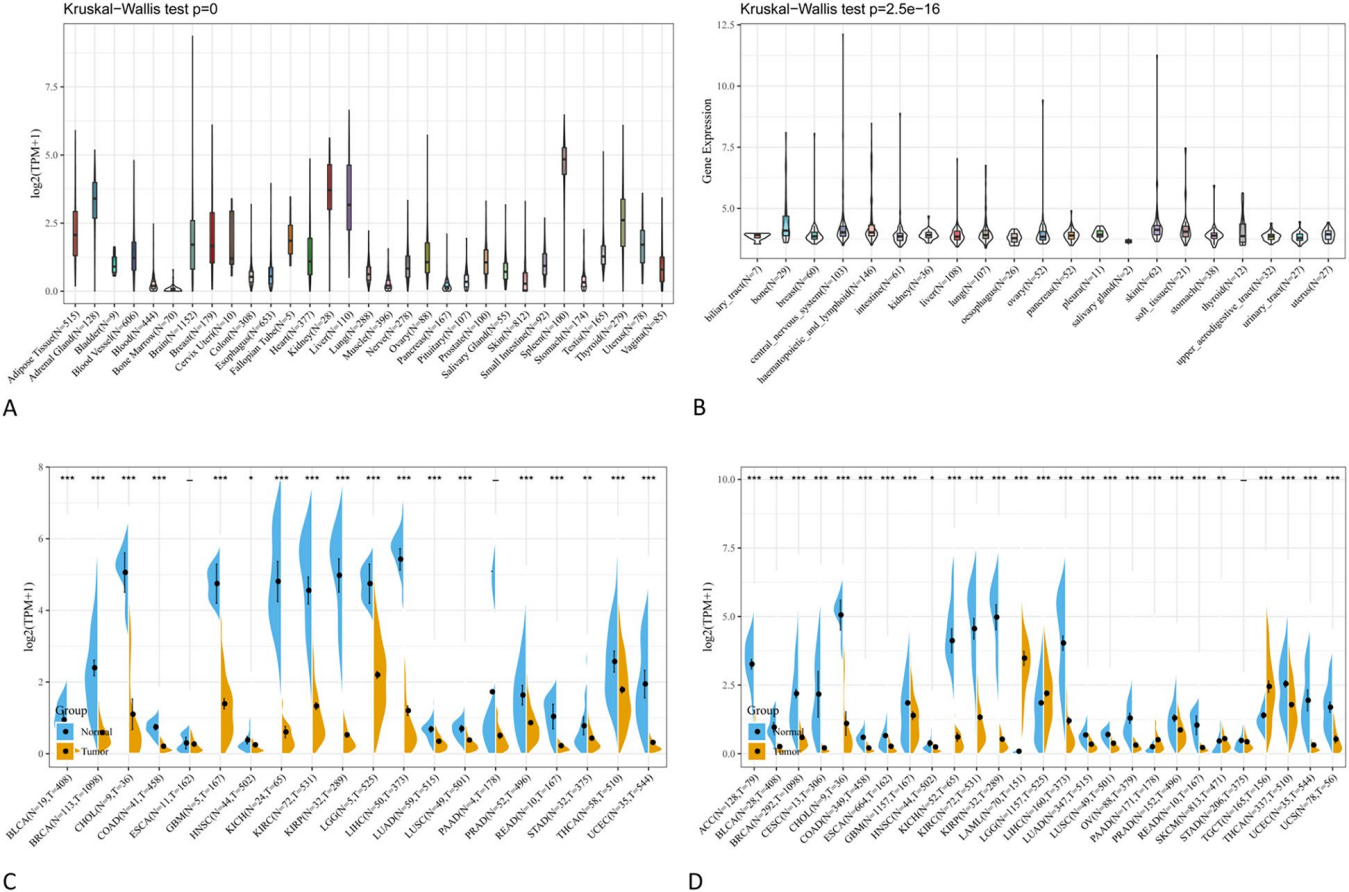
The expression of CRHBP in normal and malignant samples through using GTEx and TCGA datasets. (**A**) CRHBP expression in normal tissues based on the GTEx databases; (**B**) CRHBP expression in normal tissues based on the TCGA databases; (**C**) CRHBP expression in 20 tumor tissues and corresponding normal tissues based on the TCGA database; (**D**) CRHBP expression in 27 tumor tissues and corresponding normal tissues based on the combined database of TCGA and GTEx datasets.

**Figure 2:**
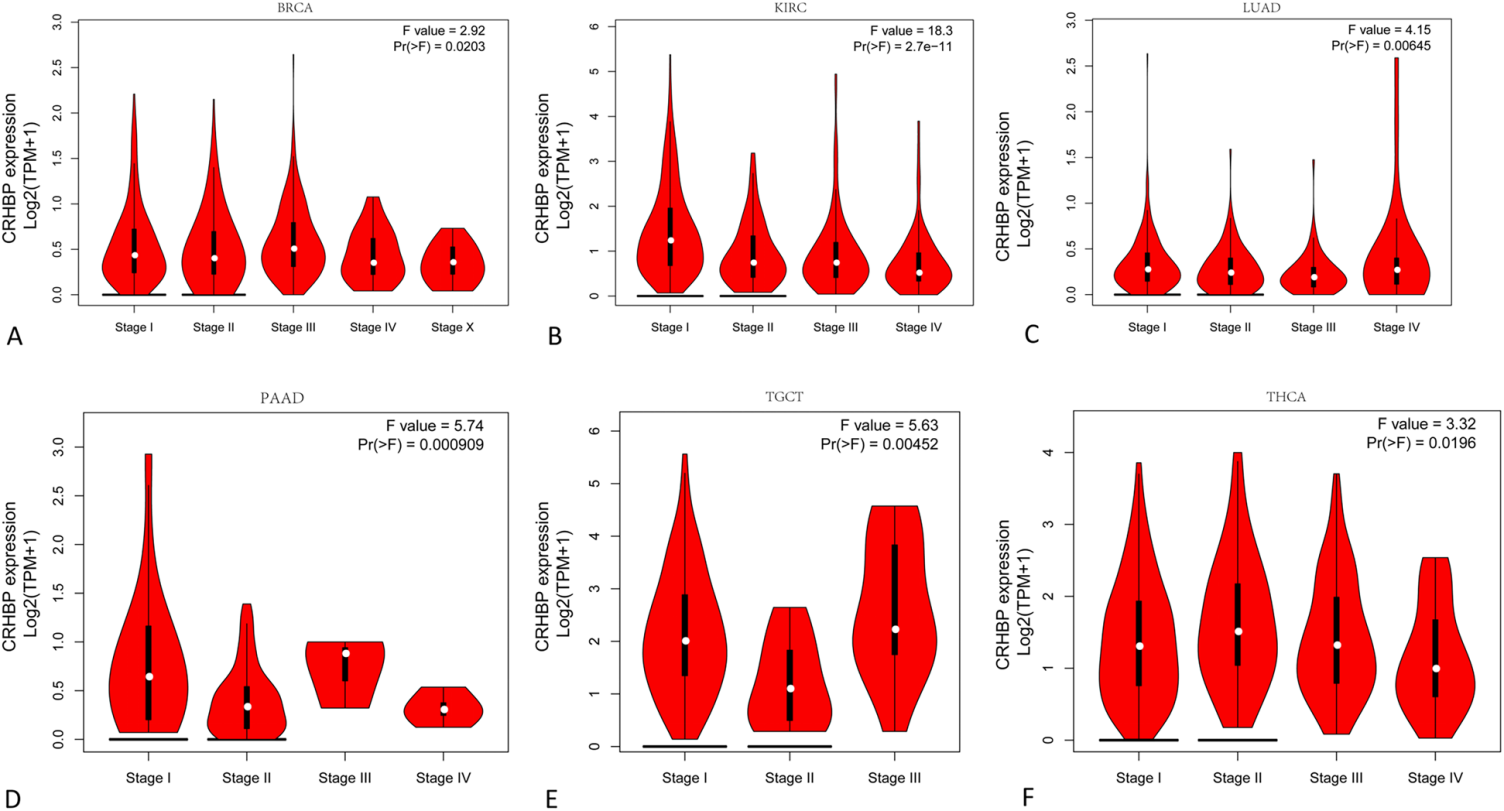
Statistically significant alterations in CRHBP expression levels across the major pathogenic phases. There were significant differences in CRHBP expression between the main pathological stages of BRCA (stages I-V), KIRC, LUAD, PAAD, THCA (stages I-IV), and TGCT (stages I, II, and III) (all p < 0.05).

Figure 1 shows the outcomes of our analysis of the expression of CRHBP in normal and malignant samples through using GTEx and TCGA datasets. We analyzed the levels of CRHBP expression in 20 tumor and normal tissues utilizing TCGA database, and we carried out a similar study for 27 tumor types using the combined TCGA and GTEx datasets. In 17 tumor samples, including lung adenocarcinoma (LUAD), breast invasive carcinoma (BRCA), cholangiocarcinoma (CHOL), kidney chromophobe (KICH), lung squamous cell carcinoma (LUSC), prostate adenocarcinoma (PRAD), rectum adenocarcinoma (READ), colon adenocarcinoma (COAD), glioblastoma multiforme (GBM), kidney renal papillary cell carcinoma (KIRP), brain lower grade glioma (LGG), bladder urothelial carcinoma (BLCA), kidney clear cell carcinoma (KIRC), thyroid carcinoma (THCA), and uterine corpus endometrial carcinoma (UCEC) (p < 0.001), head and neck squamous cell carcinoma (HNSC) (p < 0.05), and stomach adenocarcinoma (STAD) (p < 0.01), the expression levels of CRHBP were significantly lower than those in the comparable normal tissues. While based on the integrated database, CRHBP expression was significantly lower in 21 species including uterine serous carcinoma (USC), LUAD, LUSC, UCEC, CHOL, COAD, ovarian serous cystadenocarcinoma (OV), PRAD, BRCA, esophageal carcinoma (ESCA), GBM, KICH, KIRC, READ, cervical squamous cell carcinoma and endocervical adenocarcinoma (CESC), adrenocortical carcinoma (ACC), BLCA, KIRP, liver hepatocellular carcinoma (LIHC), THCA (p < 0.001) and HNSC (p < 0.05), but significant higher expression was found in acute myeloid leukemia (LAML), LGG, pancreatic adenocarcinoma (PAAD), testicular germ cell tumor (TGCT) (p < 0.001) and skin cutaneous melanoma (SKCM) (p < 0.01). Moreover, we found statistically significant alterations in CRHBP expression levels across the major pathogenic phases of BRCA (stages I–V), KIRC, LUAD, PAAD, THCA (stages I–IV), and TGCT (stages I–III) (Fig. 2, all *p* < 0.05). CRHBP expression level also shown significant statistical difference between different pathological stages in the whole pan-cancer (see Supplementary Fig. S1 online).

### The CRHBP may be used as a universal biomarker for cancer survival prediction

**Figure 3:**
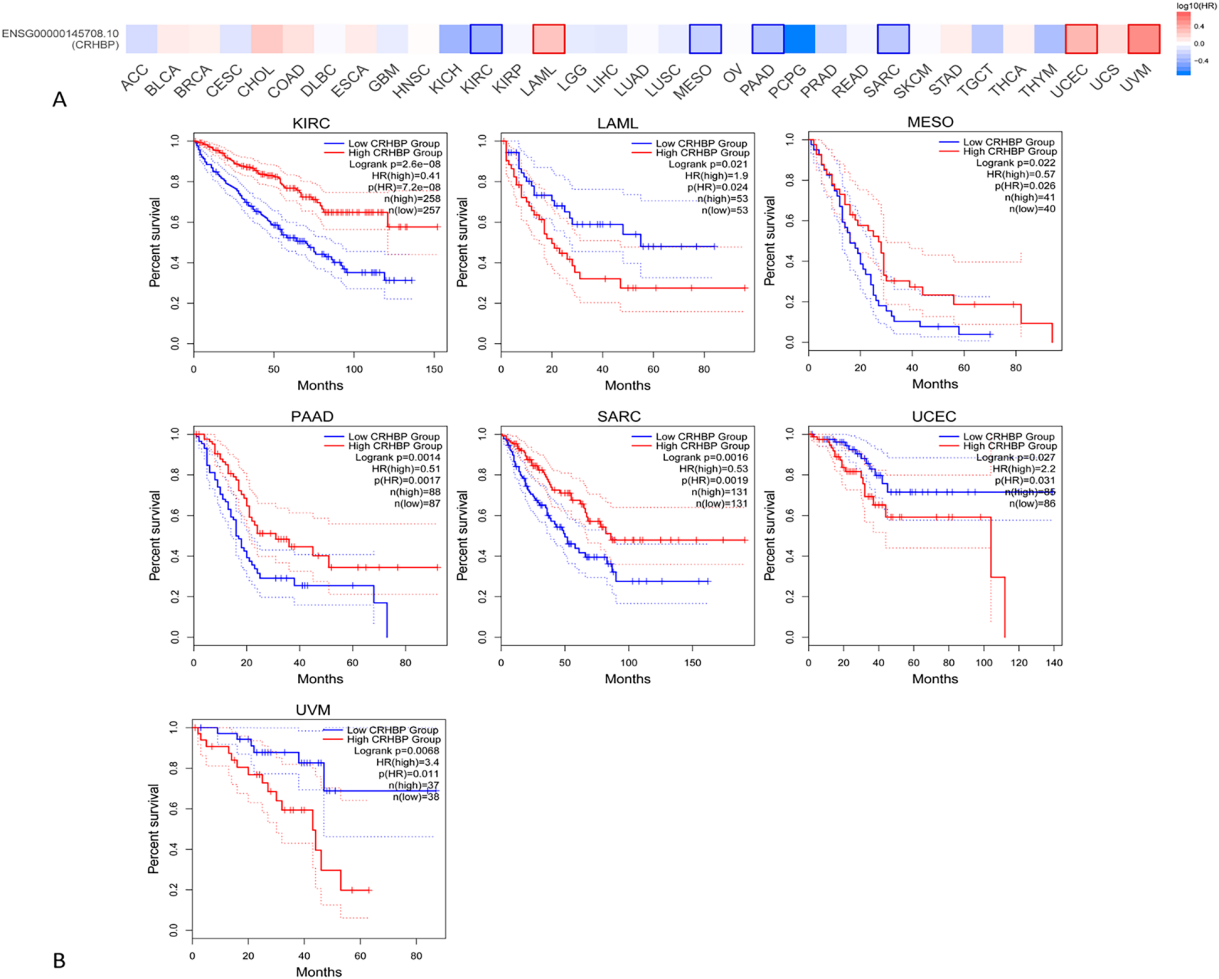
The association between CRHBP expression and overall survival (OS). OS analysis showed that high expression of CRHBP was associated with poor prognosis in LAML (p = 0.024), UCEC (p = 0.031), UVM (p = 0.011), while low expression of CRHBP was associated with poor prognosis in KIRC (p < 0.001), MESO (p = 0.026), PAAD (p = 0.0017), SARC (p = 0.0019).

**Figure 4:**
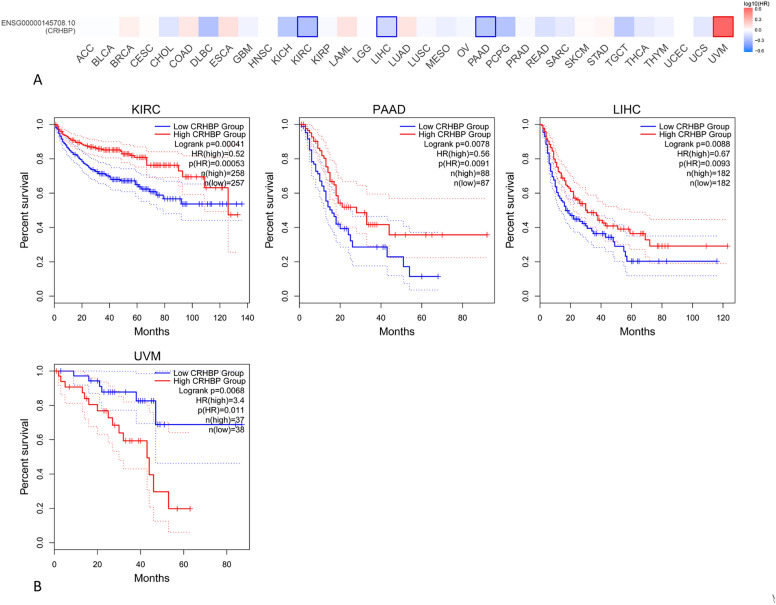
The association between CRHBP expression and disease-free survival (DFS). DFS analysis showed that low expression of CRHBP in KIRC (p = 0.00053), PAAD (p = 0.0091), and LIHC was associated with poor prognosis, while high expression of CRHBP in UVM (p = 0.011) was associated with poor prognosis.

Using the TCGA GEPIA2 tool, we investigated the association between CRHBP expression and overall survival (OS) and disease-free survival (DFS) in a variety of malignancies. Low CRHBP expression was linked to a bad prognosis in the following cancers: KIRC (*p* < 0.001), MESO (*p* = 0.026), PAAD (*p* = 0.0017), and SARC (*p* = 0.0019), whereas high CRHBP expression was linked to a poor prognosis in the following cancers: LAML (*p* = 0.024), UCEC (*p* = 0.031), and UVM (*p* = 0.011). According to the DFS analysis, whereas high CRHBP expression in UVM (p = 0.011) was associated with a poor prognosis, low CRHBP expression in KIRC (*p* = 0.00053), PAAD (*p* = 0.0091), and LIHC (*p* = 0.0093) was also associated with a poor prognosis. According to the aforementioned research, the expression of CRHBP is correlated with the prognosis of various cancers. Figures 3, 4 provide Kaplan–Meier curves with statistically significant findings.

### Correlations between CRHBP expression levels and immunological checkpoint gene markers

**Figure 5:**
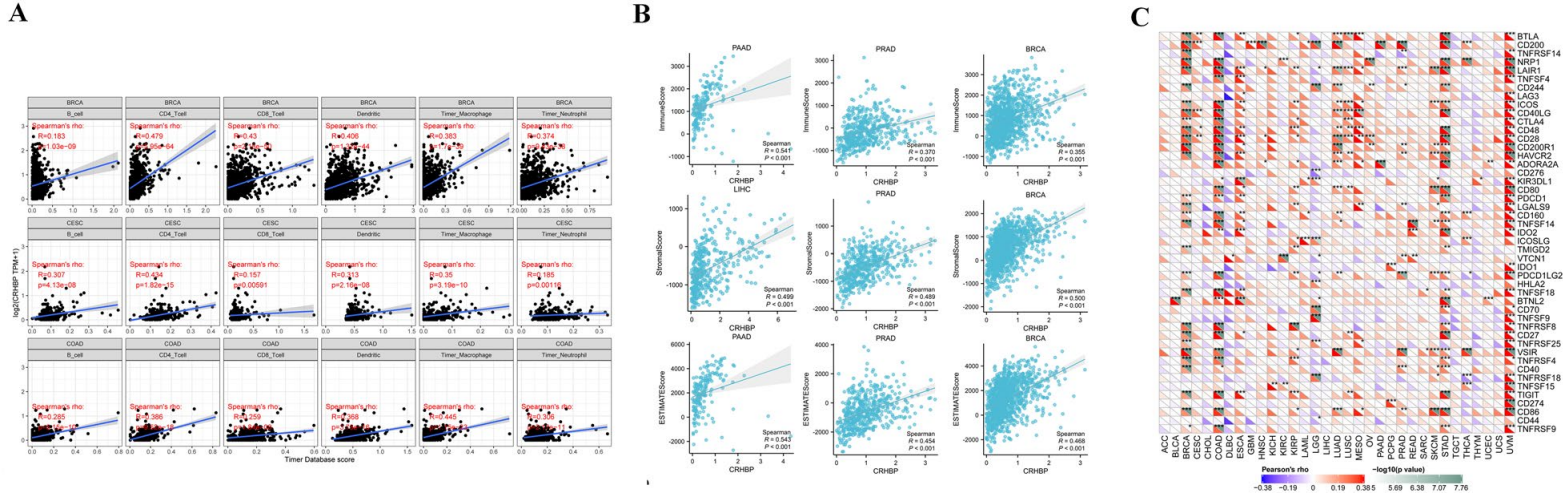
Correlations between CRHBP expression levels and tumor immunity. (**A**) TIMER method showed a positive correlation between CRHBP and the abundance of all six immune cells, including B cells, CD4 + T cells, CD8 + T cells, neutrophils, macrophages, and dendritic cells in BRCA, CESC and COAD, which were the top three tumor groups; (**B**) ESTIMATE method showed that the top three tumors most significantly correlated with expression of CRHBP were LIHC, PRAD and BRCA (StromalScore), PAAD, PRAD and BRCA (ImmuneScore), PAAD, PRAD and BRCA (ESTIMATEScore), respectively; (**C**) The correlation of CRHBP expression with multiple immune checkpoint markers in different cancer types.

We compared the expression of CRHBP with the level of immune infiltration in each type of tumor, and TIMER results revealed a positive correlation between CRHBP and the number of all six immune cells, including B cells, CD4 + T cells, CD8 + T cells, neutrophils, macrophages, and dendritic cells in BRCA, CESC, and COAD, which were the top three tumor groups (Fig. 5A).

Immune and stromal scores were independently calculated using the ESTIMATE method. In order, LIHC, PRAD and BRCA (StromalScore), PAAD, PRAD and BRCA (ImmuneScore), and PAAD, PRAD and BRCA (ESTIMATEScore) were the top three tumors with the greatest levels of CRHBP expression (Fig. 5B).

We evaluated the connection of CRHBP expression with several immune checkpoint markers in various cancer types to further explore the potential of CRHBP in immunotherapy (Fig. 5C). We discovered that the expression levels of the majority of immunological checkpoint markers in various cancers were favorably connected with the expression levels of CRHBP, while just a few were negatively correlated. Among them, the expression levels of the majority of immune checkpoint markers were highly positively correlated with UYM, STAD, COAD, and BRCA.

### Several subtypes of malignancies' immunological and molecular systems express CRHBP differently

**Figure 6:**
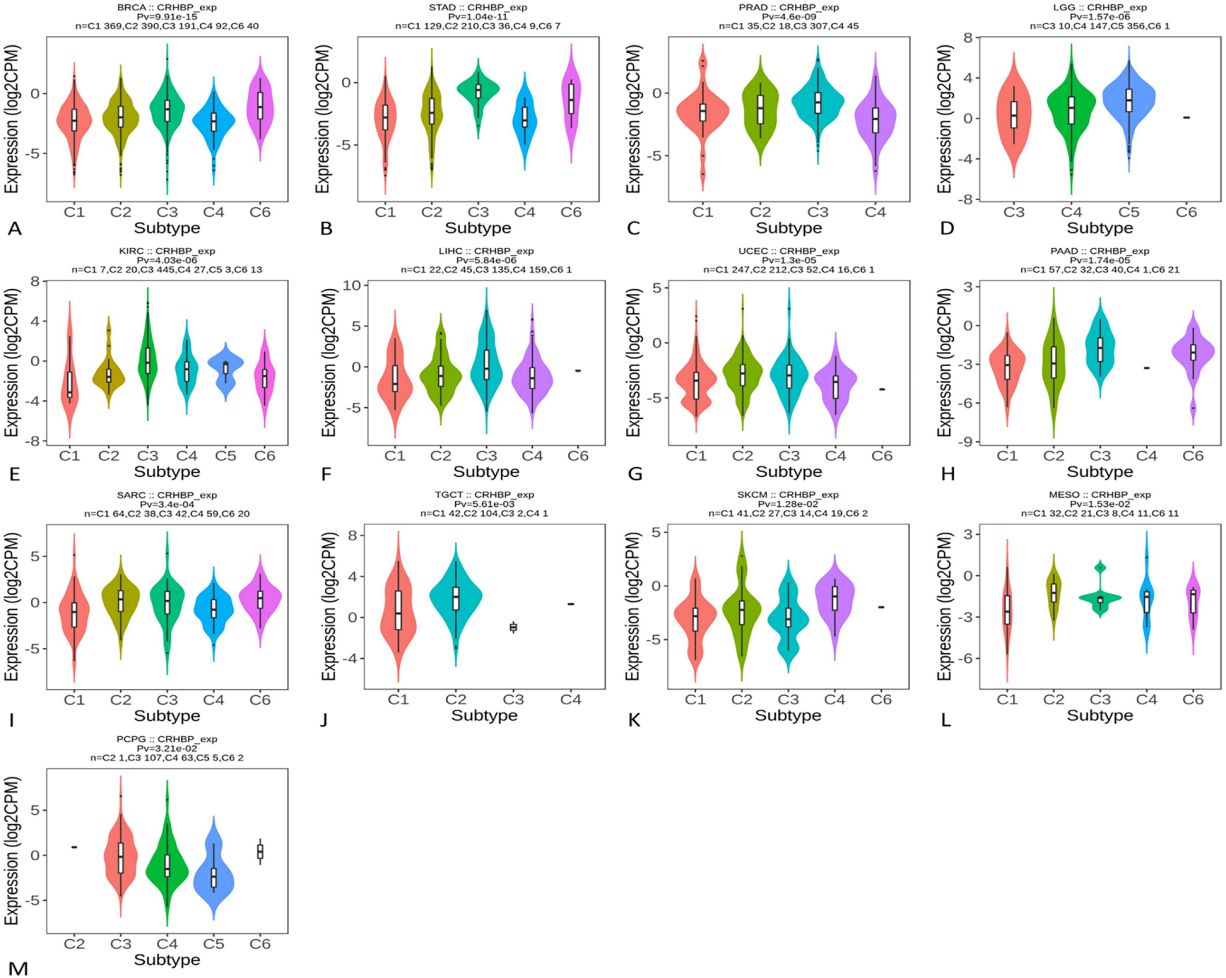
The expression of CRHBP was statistically different in several immunological subtypes. The expression of CRHBP in BRCA, STAD, PRAD, LGG, KIRC, LIHC, UCEC, PAAD, SARC, TGCT, SKCM, MESO, PCPG was statistically different in different immune subtypes (C1: wound healing; C2: IFN-γ predominant; C3: inflammation; C4: lymphocyte exhaustion; C5: immune silencing; C6:TGF-b predominant).

**Figure 7:**
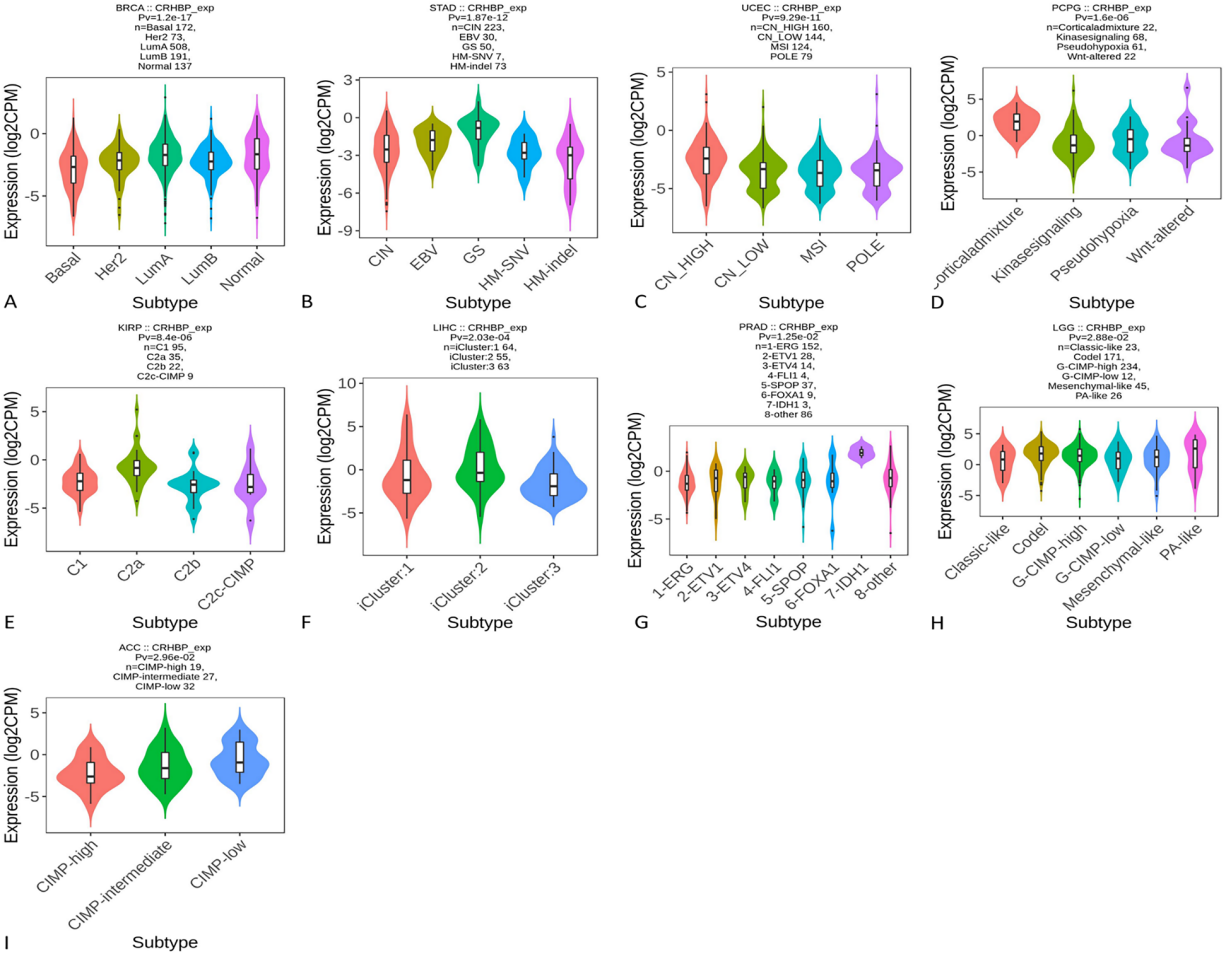
The expression of CRHBP was statistically different in several molecular subtypes. There were significant differences in CRHBP expression for different molecular subtypes of BRCA, STAD, UCEC, PCPG, KIRP, LIHC, PRAD, LGG, and ACC.

Using the TISIDB database, we further investigated how the expression of CRHBP varied across various immunological and molecular subtypes of tumors. Six immune subtypes were examined, including C1 (wound healing), C2 (IFN-predominant), C3 (inflammation), C4 (lymphocyte fatigue), C5 (immune silence), and C6 (inflammation) (TGF-b predominant). The expression of CRHBP was statistically different in several immunological subtypes, as shown in Fig. 6, in BRCA, STAD, PRAD, LGG, KIRC, LIHC, UCEC, PAAD, SARC, TGCT, SKCM, MESO, and PCPG. For various molecular subtypes of BRCA, STAD, UCEC, PCPG, KIRP, LIHC, PRAD, LGG, and ACC, there were also notable variations in CRHBP expression (Fig. 7). We may infer from the data above that the immunological and molecular subtypes of pan-cancer express CRHBP differently.

### CRHBP expression correlates with tumor mutational load (TMB) and microsatellite instability (MSI)

**Figure 8:**
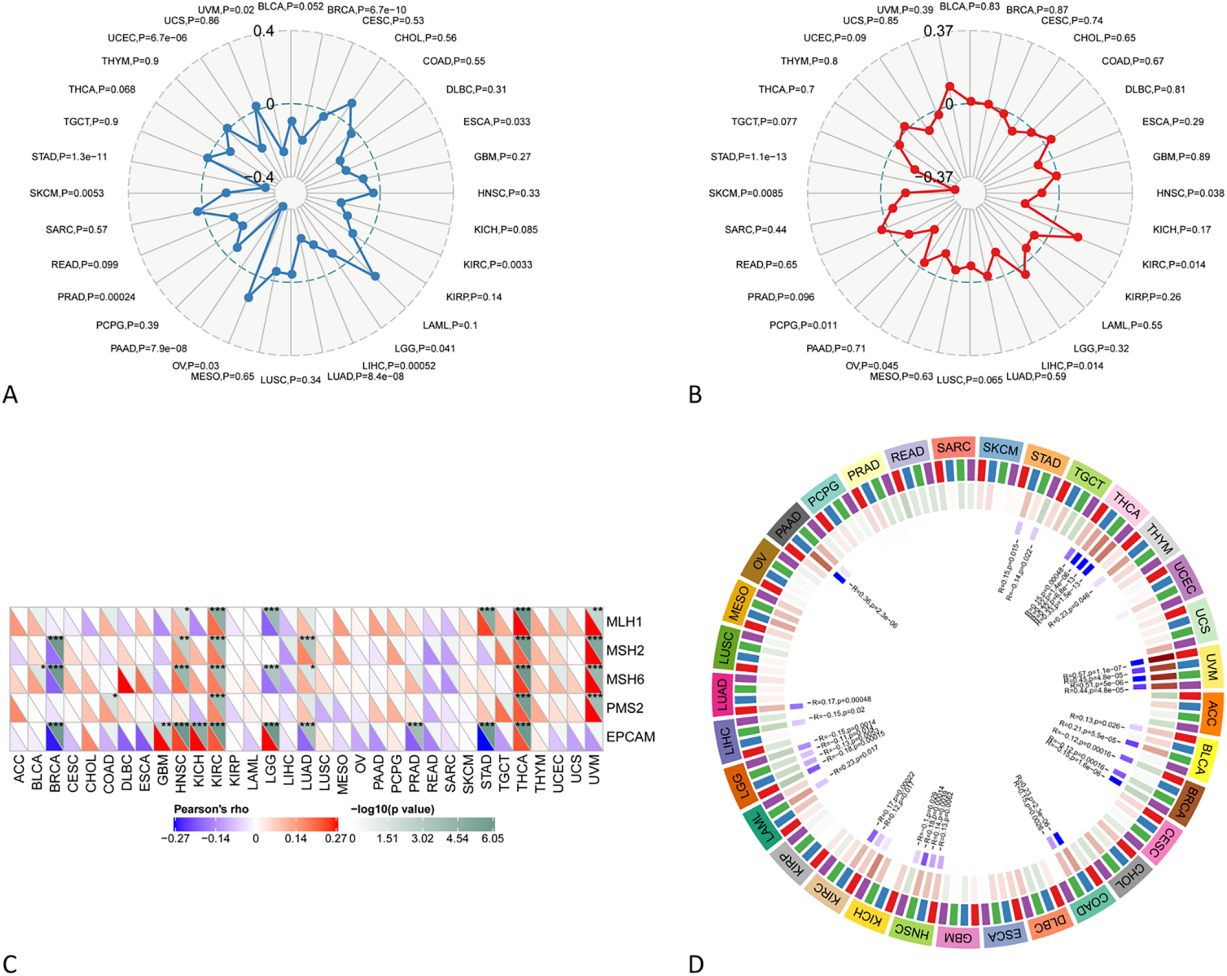
CRHBP expression correlates with tumor mutational load (TMB) and microsatellite instability (MSI). (**A**) The expression of CRHBP in OV (p = 0.03) was positively correlated with TMB, while that in BRCA, KIRC, LIHC, LUAD, PAAD, PRAD, SKCM, STAD, UCEC was strongly negatively correlated with TMB (p < 0.001); (**B**) The expression of CRHBP in STAD, SKCM (p < 0.001) and PCPG, OV, LIHC, KIRC, HNSC (p < 0.05) was negatively correlated with MSI, while that in KIRC (p = 0.014) was positively correlated with MSI; (**C**) CRHBP expression was correlated with MMR genes (MLH1, MSH2, MSH6, EPCAM and PMS2) in 13 cancers including BLCA, BRCA, COAD, GBM, HNSC, KICH, KIRC, LGG, LUAD, PRAD, STAD, THCA, and UVM; (**D**) CRHBP was highly correlated with four DNA methyltransferases (DNMT1, DNMT2, DNMT3A, and DNMT3B) in various cancers such as PAAD, LUAD, LGG, etc.

We also assessed the relationship between CRHBP expression and TMB and MSI. The expression of CRHBP in OV (*p* = 0.03) was positively correlated with TMB, while that in BRCA, KIRC, LIHC, LUAD, PAAD, PRAD, SKCM, STAD, UCEC was strongly negatively correlated with TMB (*p* < 0.001, Fig. 8A). In STAD, SKCM (*p* < 0.001) and PCPG, OV, LIHC, KIRC, HNSC (*p* < 0.05), the expression of CRHBP was negatively correlated with MSI, while in KIRC (*p* = 0.014), it was positively correlated with MSI (Fig. 8B).

### CRHBP expression correlates with MMR gene mutations and DNA methylation

We examined the relationship between the levels of CRHBP expression and the levels of mutations in five MMR genes (MLH1, MSH2, MSH6, PMS2, EPCAM) to identify CRHBP's possible contribution to tumor immunity. Figure 8C shows the relationship between the expression of the CRHBP gene and the MMR genes in 13 different types of cancer, including BLCA, BRCA, COAD, GBM, HNSC, KICH, KIRC, LGG, LUAD, PRAD, STAD, THCA, and UVM. We also explored into the connection between CRHBP and four different types of DNA methylation, and the findings revealed that in many malignancies, including PAAD, LUAD, LGG, etc., CRHBP was strongly connected with four different types of DNA methyltransferases (DNMT1, DNMT2, DNMT3A, and DNMT3B) (Fig. 8D). These findings imply that CRHBP could influence DNA methylation to control tumor development.

### Relationship between CRHBP expression levels and drug response

**Figure 9:**
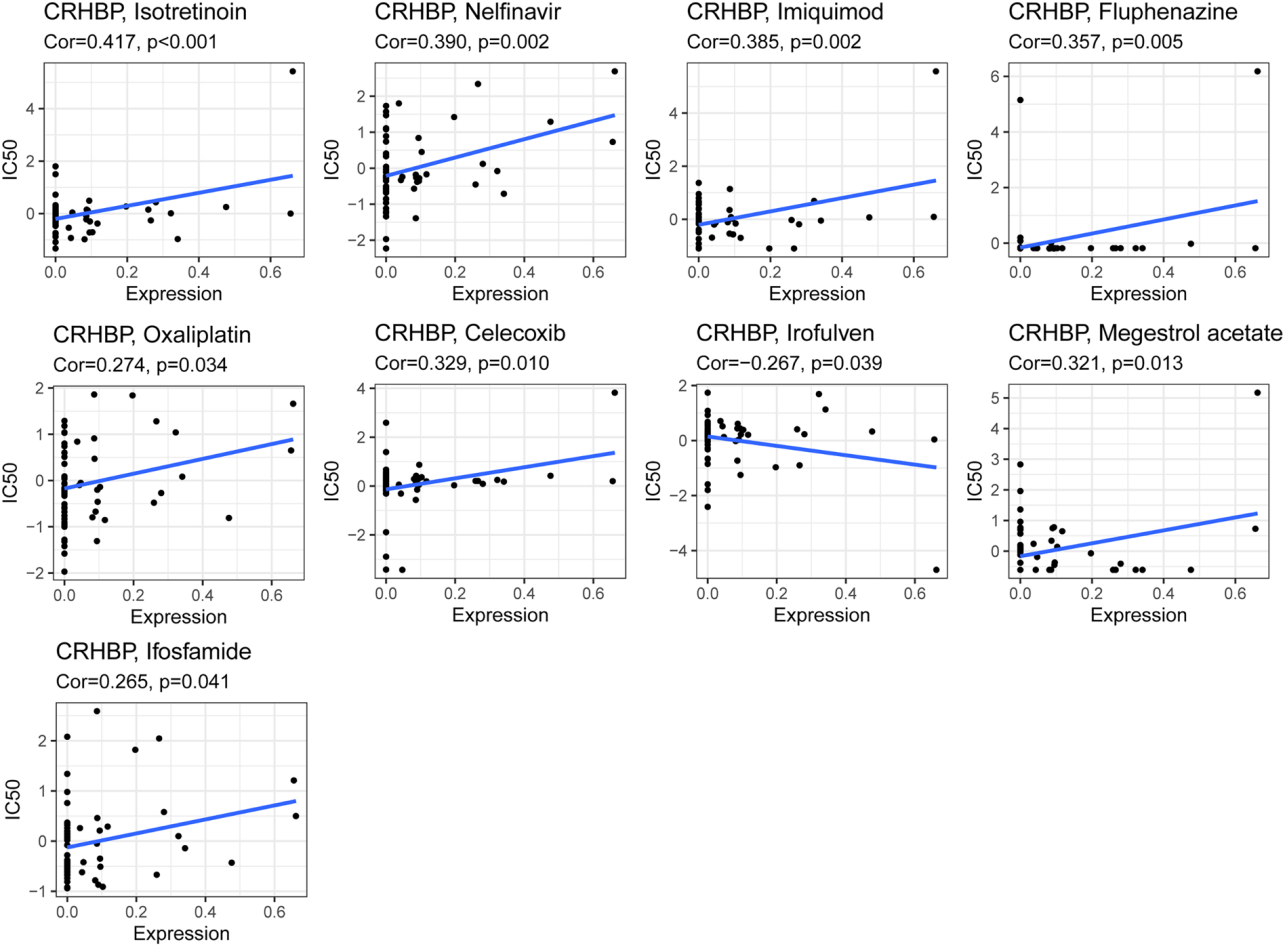
The relationship between CRHBP expression and predicted drug responses. There were a significant positive correlation between CRHBP expression levels and responses to 8 drugs including Isotretinoin, Nelfinavir, Imiquimod, Fluphenazine, Oxaliplatin, Celecoxib, Megestrol acetate, Ifosfamide (p < 0.05) and a negative correlation with Irofulven (p = 0.039).

Figure 9 depicts the relationship between CRHBP expression and predicted drug responses. There was a significant positive correlation between CRHBP expression levels and responses to 8 medications (p < 0.05), including Isotretinoin, Nelfinavir, Imiquimod, Fluphenazine, Oxaliplatin, Celecoxib, Megestrol acetate, Ifosfamide (p < 0.05), as well as a negative correlation with Irofulven (p = 0.039).

### Functional analysis based on gene pooling enrichment analysis

**Figure 10:**
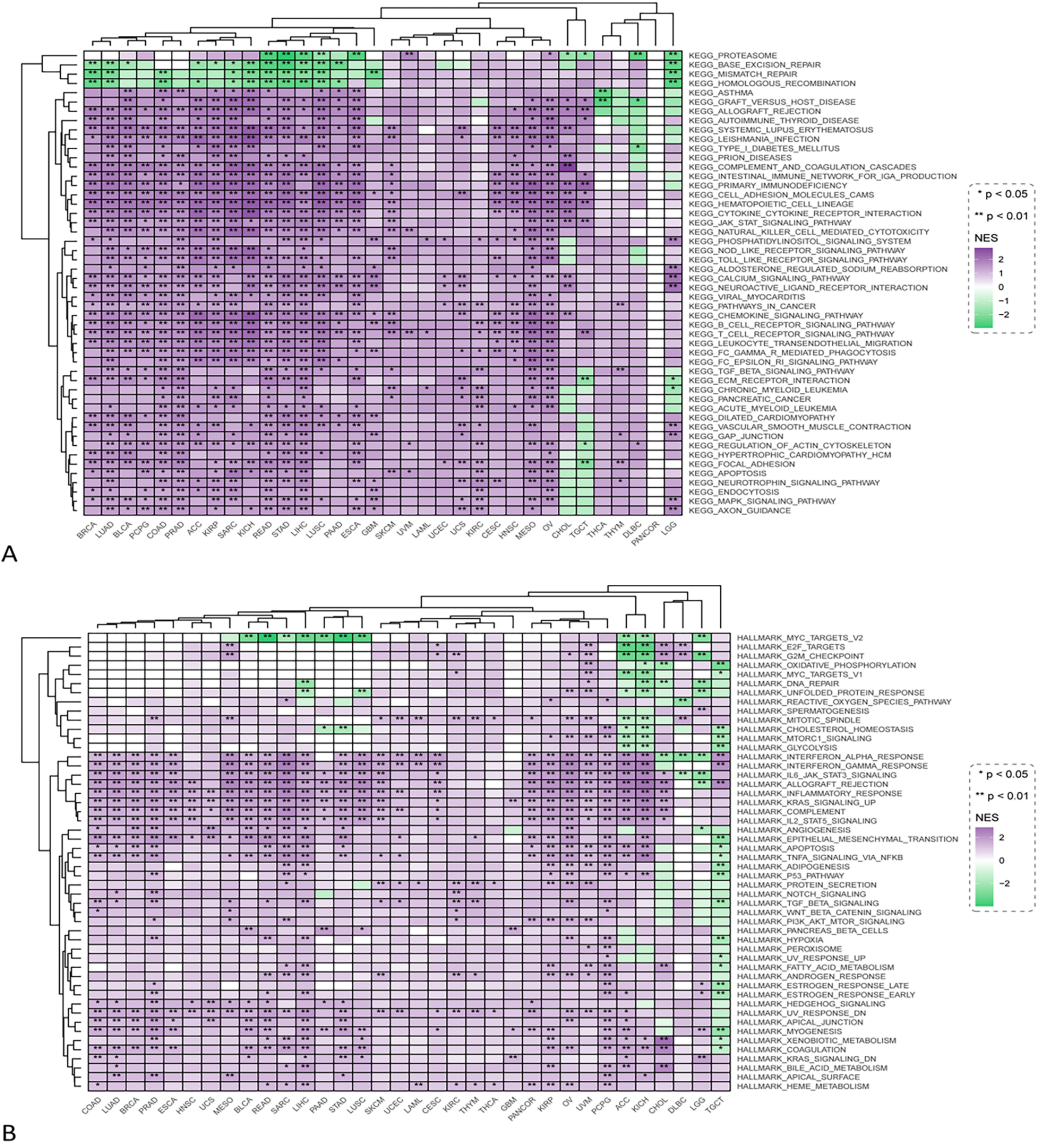
The feature enrichment of high and low CRHBP expression. (**A**) The KEGG enrichment term showed that high expression of CRHBP was mainly associated with multiple signaling pathways including JAK/STAT, NOD-like receptor, Toll-like receptor, and B/T cell receptor signaling pathways, while low expression of CRHBP was mainly associated with proteasome, base-excision repiar, mismatch repair, and homologous recombination signaling pathways; (**B**) The results of HALLMARK terms showed that the low expression of CRHBP was mainly associated with Myc targets V2, E2f targets and G2M checkpoint signaling pathway, while the high expression of CRHBP was associated with Interferon alpha/gamma respose, IL-6/JAK/STAT3, IL2/STAT5, NOTCH and many other signaling pathways.

Using the GSEA approach, we were able to determine the feature enrichment of high and low CRHBP expression (Fig. 10). The KEGG enrichment analysis revealed that the signaling pathways JAK/STAT, NOD-like receptor, Toll-like receptor, and B/T cell receptor were primarily associated with CRHBP's high expression, whereas the signaling pathways proteasome, base-excision repair, mismatch repair, and homologous recombination were primarily associated with CRHBP's low expression. According to the results of HALLMARK terms, CRHBP's low expression was primarily linked to Myc targets V2, E2f. targets, and the G2M checkpoint signaling pathway, whereas CRHBP's high expression was linked to the Interferon alpha/gamma response, IL-6/JAK/STAT3, IL2/STAT5, NOTCH, and numerous other signaling pathways. In addition, we complementarily construct a protein interaction network for CRHBP (see Supplementary Fig. S2A online). Based on the protein interaction network, we further evaluate the relationship between the GSVA score and the activity of different tumor-related pathways (see Supplementary Fig. S2B online). The results of correlation analysis showed that CRHBP can inhibit the EMT and cell cycle pathway in LIHC, supporting the results of subsequent experiment. However, the opposite result was obtained in cell apoptosis, which may be due to the heterogeneity of tumor tissue. In addition, different data processing methods in different databases will also lead to the heterogeneity of analysis results. This also indicated that the results of bioinformatics at the functional level do require experimental verification in cell and molecular biology.

### Univariate and multivariate Cox regression analysis showed that CRHBP expression was an independent prognostic factor for LIHC

We used univariate Cox regression analysis in the LIHC cohort of TCGA database to evaluate the prognostic significance of common clinical characteristics (age, gender, tumor grade, stage) and CRHBP expression level. The results showed that only tumor stage and CRHBP expression level had independent prognostic value. After adjusting for age, gender and tumor grade, multivariate Cox regression analysis showed that tumor stage and CRHBP expression were still independent prognostic factors for LIHC (Table 1). This suggests that CRHBP may be an important prognostic indicator of LIHC, which is worth further exploration.

**Table 1:** Cox regression analysis of the prognostic value of CRHBP and clinical pathological characteristics in the LIHC cohort.

Characteristics	Total (N)	Univariate analysis		Multivariate analysis	
		Hazard ratio (95% CI)	P value	Hazard ratio (95% CI)	P value
Age	373				
≤ 60	177	Reference			
> 60	196	1.205 (0.850–1.708)	0.295		
Gender	373				
Male	252	Reference			
Female	121	1.261 (0.885–1.796)	0.200		
Histologic grade	368				
G1 & G2	233	Reference			
G3 & G4	135	1.091 (0.761–1.564)	0.636		
Pathologic stage	349				
Stage I & Stage II	259	Reference		Reference	
Stage III & Stage IV	90	2.504 (1.727–3.631)	< 0.001	2.433 (1.676–3.531)	< 0.001
CRHBP	373	0.852 (0.737–0.985)	0.031	0.842 (0.714–0.991)	0.039

### CRHBP expression is down-regulated in human LIHC

**Figure 11:**
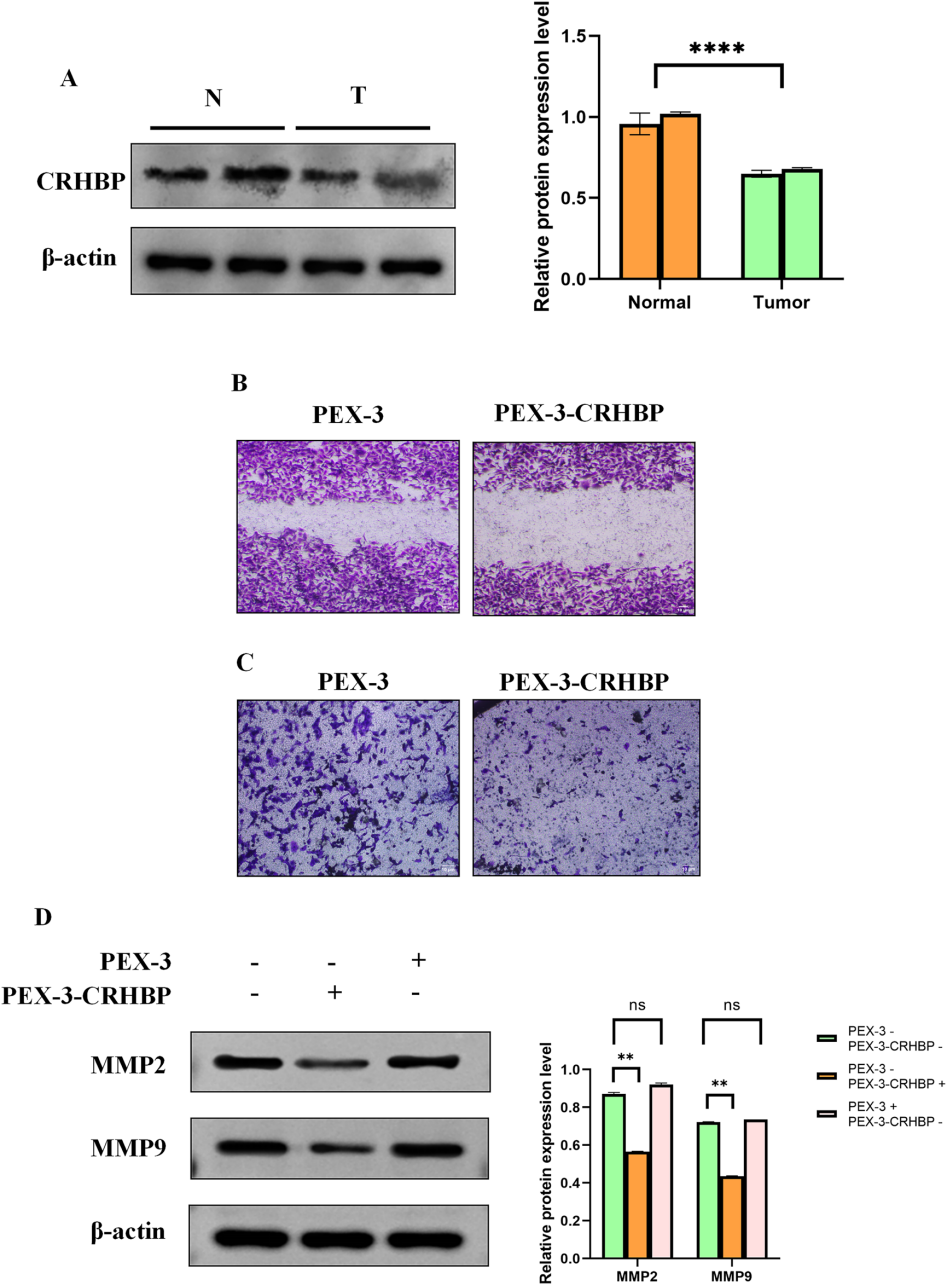
Effect of CRHBP on proliferation and apoptosis of LIHC cells. (**A**) The expression level of CRHBP was down-regulated in LIHC tissues compared to the adjacent tissues; (**B**) The EdU staining results showed that transfection of PEX-3-CRHBP significantly reduced the proliferation of HepG2 cells; (**C**) The JC-1 staining showed that the transfection of PEX-3-CRHBP promoted the apoptosis of HepG2 cells; (**D**) Western blotting results showed that PEX-3-CRHBP increased the expression level of Bax and decreased the expression levels of Bcl-2 and PCNA in HepG2 cells.

Based on the findings of Western blotting, we determined that the degree of expression for CRHBP was lowered in LIHC tissues relative to the surrounding tissues (Fig. 11A).

### Effect of CRHBP on proliferation and apoptosis of LIHC cells

We transfected HepG2 cells with PEX-3-CRHBP to boost CRHBP expression. The EdU staining findings revealed that transfection of PEX-3-CRHBP greatly decreased the proliferation of HepG2 cells (Fig. 11B), and the JC-1 staining results demonstrated that transfection of PEX-3-CRHBP increased the apoptosis of LIHC cells (Fig. 11C). Simultaneously, western blotting findings revealed that PEX-3-CRHBP decreased the expression of B-cell lymphoma-2 (Bcl-2) while increasing the expression of Bcl2-associated X protein (Bax) and proliferating cell nuclear antigen (PCNA) in LIHC cells (Fig. 11D). The previous studies suggested that CRHBP may inhibit HepG2 cell proliferation while inducing apoptosis.

### Effect of CRHBP on invasion and migration of LIHC cells

**Figure 12:**
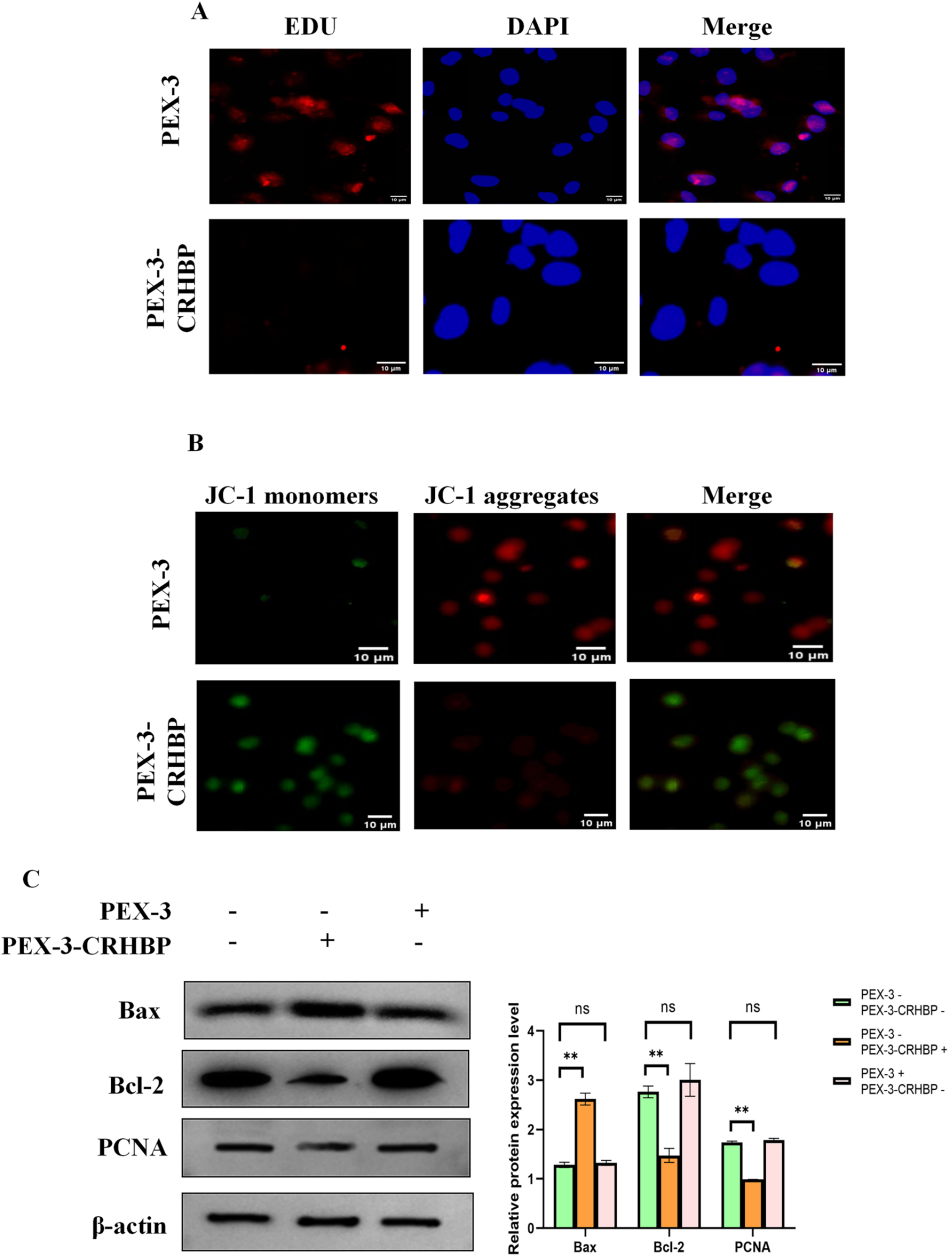
Effect of CRHBP on invasion and migration of LIHC cells. (**A**,**B**) Wound-healing and transwell experiments showed that overexpression of CRHBP significantly reduced the invasive and migratory abilities of HepG2 cells, respectively; (**C**) Western blotting results showed that PEX-3-CRHBP increased the expression level of Bax and decreased the expression levels of MMP2 and MMP9 in HepG2 cells.

To investigate the possible involvement of CRHBP in controlling the invasiveness and migration of LIHC cells, we conducted wound-healing and transwell assays. According to the findings, CRHBP overexpression dramatically decreased the invasive and migratory properties of HepG2 cells (Fig. 12A,B). Meanwhile, changes in MMP-2 and MMP-9 protein expression levels correlated with wound healing and transwell test results (Fig. 12C). As a consequence, we may infer that CRHBP can inhibit in vitro HepG2 cell invasion and migration.

## Discussion

Our investigation discovered that CRHBP expression levels were much lower in the majority of cancer types compared to normal tissues, but considerable high expression was also detected in the cancer types LAML, LGG, PAAD, and TGCT, which was consistent with previous findings^3,15,19^. CRHBP expression was shown to be favorably linked with the expression level of many ICPs, including LAG3, CTLA4, PD-1, CD28, CD48, PD-L1, PD-L2, and others. The GSEA approach suggested that signaling pathways such as IFN/respose, IL-6/JAK/STAT3, IL2/STAT5, and B/T cell receptor were the main functional enrichment items involved in the high expression of CRHBP, whereas G2M checkpoint, base-excision repair, mismatch repair, and homologous recombination were the main functional enrichment items involved in the low expression of CRHBP. These results were congruent with those of prior animal and cell-based investigations^4,6,12^

CRHBP expression varied according to the clinical phases of several malignancies as well as according to the various immunological and molecular subtypes, and the expression of CRHBP was connected to the prognosis for survival of numerous tumors. As for these, the number of previous studies on CRHBP in cancer is limited, and only a few results are generally consistent with our findings. Wang et al. utilized a large number of high-dimensional RNA sequencing files and clinical datasets collected in the Genomic Data Commons Data Portal to systematically analyze 36 important pivotal genes associated with ovarian plasmacytic cystic adenocarcinoma (OVSC) survival prognosis by integrating co-expression network analysis and Kaplan–Meier methods. The findings revealed a connection between high CRHBP expression and increased OVSC survival (*p* = 0.016)^18^. Using clinical, transcriptomic, and clinical transcriptomic data, Deng et al. performed a multicategorical prognosis classification of lung adenocarcinoma patients and observed that reduced expression levels of CRHBP were linked with survival without disease progression (PFS, coefficient = − 0.314) and survival with disease (AWD, coefficient = 0.228)^20^. Yang et al. investigated that CRHBP expression was abnormally reduced in ccRCC samples and cell lines with a positive correlation with overall patient survival (OS) (*p* < 0.0001), and notably, They also discovered that CRHBP expression levels were closely associated with patients' clinical stage and histological grade^6^. Xia et al. discovered that low CRHBP expression indicated a poor prognosis in individuals with hepatocellular cancer (p < 0.001)^16^. Our results suggest that CRHBP is involved in the development of many different forms of cancer and has potential as a pan-cancer biomarker that may predict survival prognosis. Our studies illustrate that the expression variations and prognostic importance of CRHBP need further investigation in more cancer types.

The abundance of six immune cells, including B cells, CD4 + T cells, and CD8 + T cells, among others, as well as stromal and immunological scores in a number of malignancies, including LIHC, PRAD, BRCA, and PAAD, have all been demonstrated to be highly and positively correlated with CRHBP expression. We evaluated the immunotherapeutic potential of CRHBP and discovered that in 28 cancer types, including LIHC, THCA, LGG, and UVM, 47 common ICP genes were more or less favorably connected with CRHBP expression, and in UVM, 37 of 47 immunological checks were positively correlated with CRHBP expression. This shows that CRHBP may coordinate the activity of several ICP genes through distinct signaling pathways, making it a suitable target for enhancing the efficacy of immunotherapy; its low expression may anticipate a favorable result of ICP gene immunotherapy. In LGG, PRAD, and THCA, however, the negative connection between CRHBP and ICP genes revealed that low CRHBP expression might be predictive of inferior immunotherapeutic results when targeting ICP genes. In sum, we could speculate that CRHBP can be used as a pan-cancer biomarker to predict immunotherapy success, and perhaps as an additional target for enhancing ICP gene immunotherapy.

Moreover, TMB and MSI were linked to CRHBP expression. Human cells have a class of safety and security genes called MMR genes that are responsible for repairing DNA base mismatches. These genes can recognize and correct base pairing errors on DNA strands, ensuring the stability and consistency of DNA. Some tumor cells may accumulate base pairing errors, leading to the production of new antigens. These new antigens are called tumor specific antigens (TSA) or tumor associated antigens (TAA), and may be targets that immune cells can recognize and attack when tumor immunotherapy is in effect. Defects in mismatch repair genes may lead to the accumulation of DNA damage, thereby increasing the production of TSA and TAA, making them targets for tumor immunotherapy. MSI has been associated to mutations in the MMR gene^21,22^. Our results showed that MSI and mutations in five MMR genes (MLH1, MSH2, MSH6, EPCAM, and PMS2) were associated to CRHBP expression in the vast majority of cancer types. DNA methylation is one of the earliest and best-studied examples of epigenetic control. Its function in chromatin structure, DNA conformation, DNA stability, and DNA–protein interactions^23–25^ makes it a unique predictor of carcinogenesis. In line with the findings of a tumor-specific epigenetic research on CRHBP in renal cell carcinoma by Tezval et al. in 2016, our findings demonstrate that CRHBP is a critical epigenetic module that may regulate tumor progression by modulating DNA methylation in pan-cancers^10^.

Our investigation into the relationship between CRHBP expression and drug response also revealed a positive correlation between CRHBP expression level and tumor responses to 12 chemotherapy drugs, including isotretinoin, Nelfinavir, and imiquimod (*p* < 0.05), and a negative correlation between CRHBP expression level and tumor responses to irofulven (*p* = 0.039). We think this might help us choose a more effective drug for tumor treatment.

In addition, we found that CRHBP expression level had independent prognostic value in LIHC by univariate and multivariate Cox regression analysis. Therefore, we further used cell and molecular biology techniques to confirm the function of CRHBP in LIHC. Western blotting revealed a downregulation of CRHBP expression in LIHC tissues. Using EdU labeling, we found that CRHBP inhibited the growth of LIHC cells. JC-1 staining demonstrated that CRHBP encouraged LIHC cells to undergo apoptosis. The Wound Healing as well as Transwell experiments demonstrated that CRHBP inhibited the invasion and migration of LIHC cells. These findings corresponded with those of our bioinformatics study.

Nevertheless, our study has a few caveats. Initially, we looked at CRHBP translation products after analyzing mRNA levels, although further work is required to establish the importance of these findings. Second, external validation from other publicly available datasets is necessary to verify our present results. Third, our methodology determined that systematic biases from different databases were still in existence. Consequently, more systematic research is needed to validate the utility of CRHBP as a biomarker for immunotherapy evaluation and a possible target for anticancer treatment, and to examine the function of CRHBP in other malignancies.

## Conclusions

In conclusion, we performed the first investigation of CRHBP in pan-cancer, and the findings revealed that CRHBP expression is correlated with survival prognosis, MMR mutation, DNA methylation, immunological infiltration, ICP, TMB, MSI, and chemotherapeutic treatment response in pan-cancer. We also conducted a functional analysis based on pooled gene enrichment analysis to determine the molecular pathways associated with CRHBP. To further verify the effect of CRHBP on tumor cell biology in LIHC, we used cellular and molecular biological techniques. All of these will help us better understand CRHBP and how it contributes to carcinogenesis and how to use immunotherapy to treat tumors.

### Supplementary Information

Supplementary Information.

## Data Availability

The original contributions presented in the study are included in the article; further inquiries can be directed to the corresponding author.
