# Supplementary material for: A pan-cancer analysis uncovering the function of CRHBP in tumor immunity, prognosis and drug response: especially its function in LIHC
Source: Sci Rep. 2024 Feb 7;14:3112. doi: 10.1038/s41598-024-52387-8 (PMC10850363; doi:10.1038/s41598-024-52387-8)
Supplement: Supplementary file 1 — Supplementary Information. [file 41598_2024_52387_MOESM1_ESM.pdf]

Suppmentary Figure 1

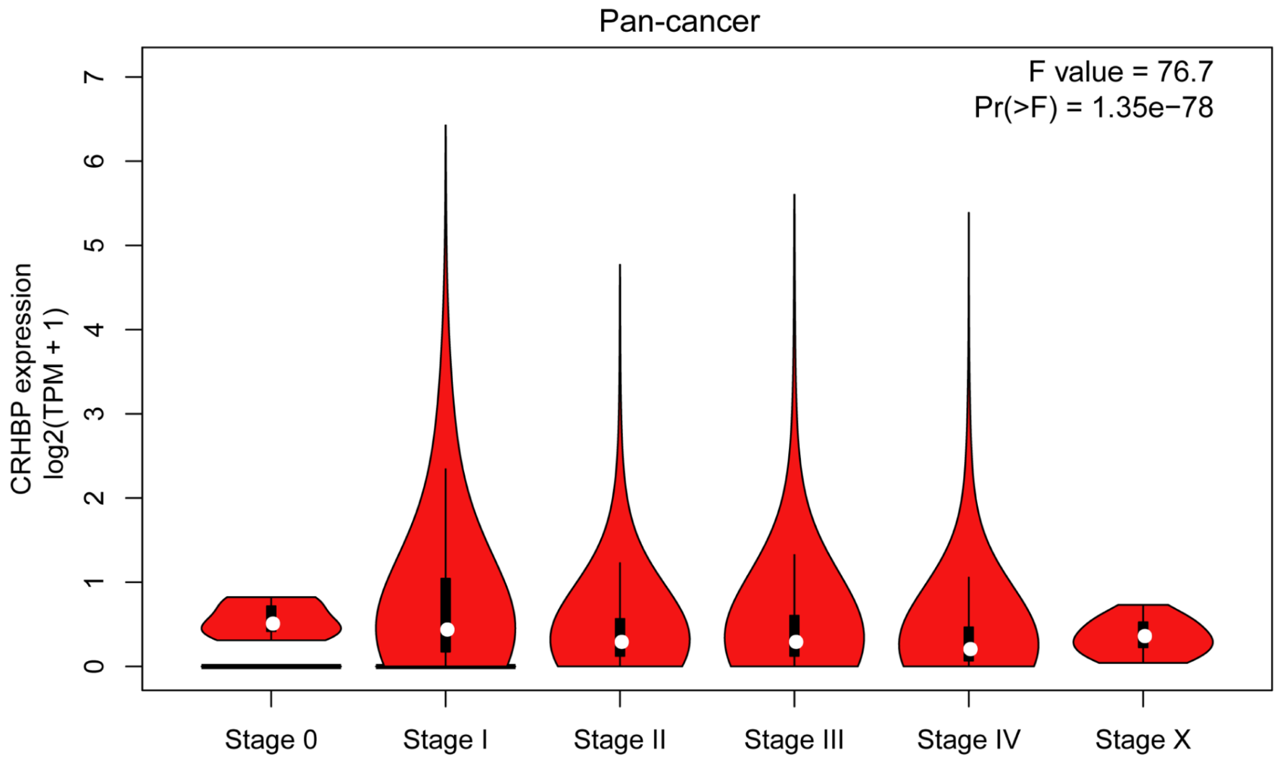

Suppmentary Figure 1 CRHBP expression level shown significant statistical difference between different pathological stages in the whole pan-cancer.

Suppmentary Figure 2

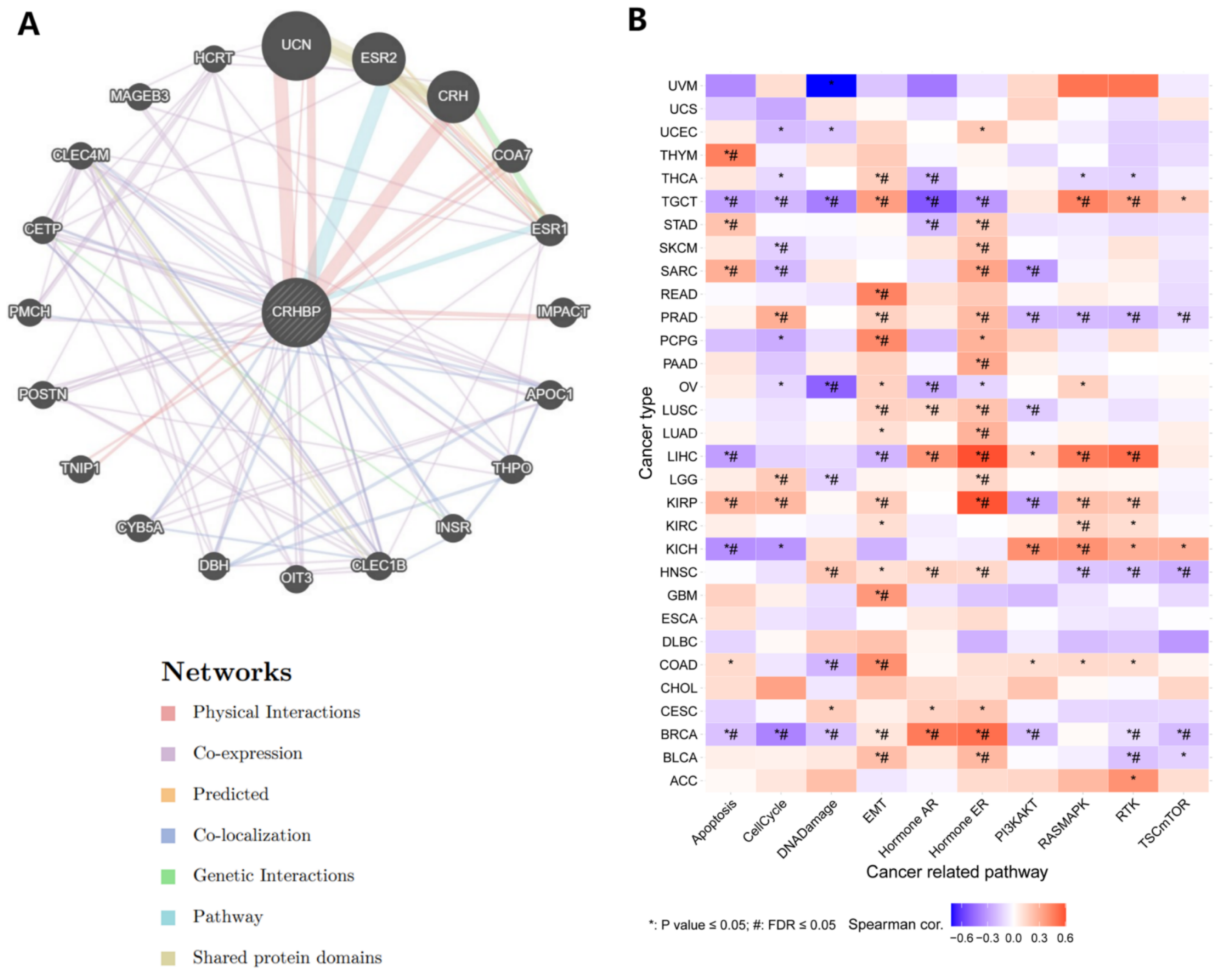

Suppmentary Figure 2 (A) Protein interaction network for CRHBP (B) Heat map reflecting the relationship between GSVA score and activity of different tumor-related pathways.

Attachment: One-to-one correspondence between blotting band and whole membrane

Figure 11 A

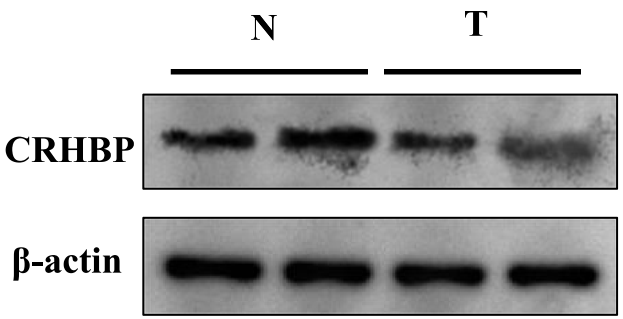

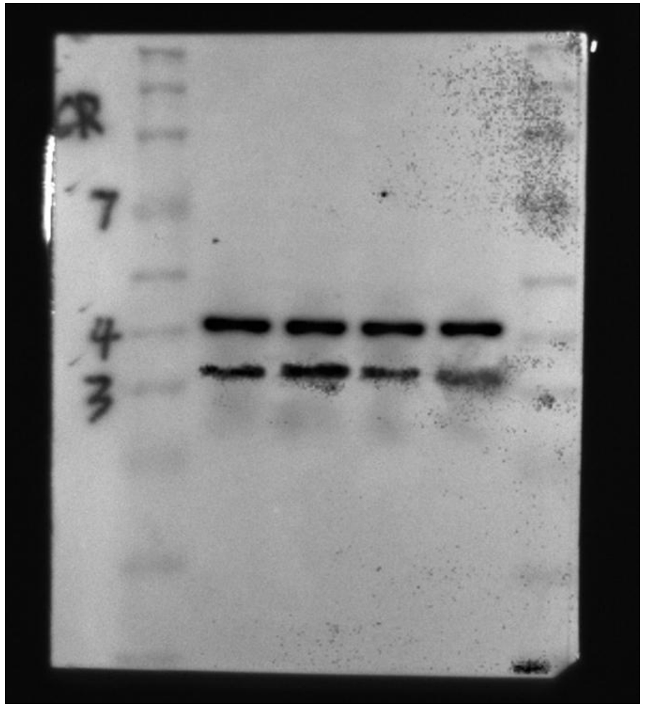

Figure 11 D

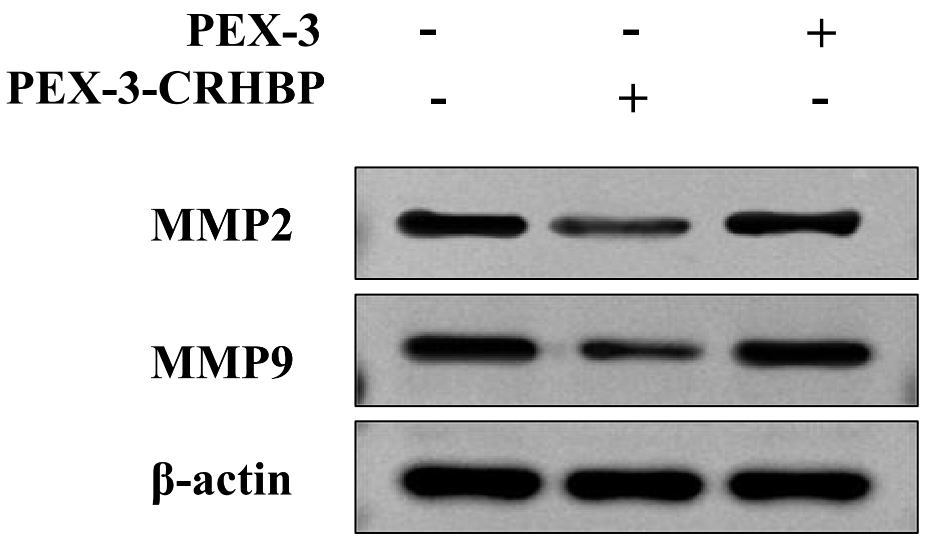

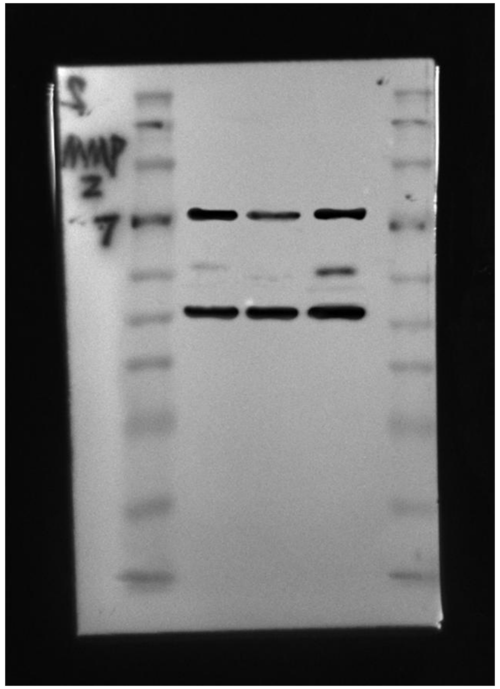

MMP2

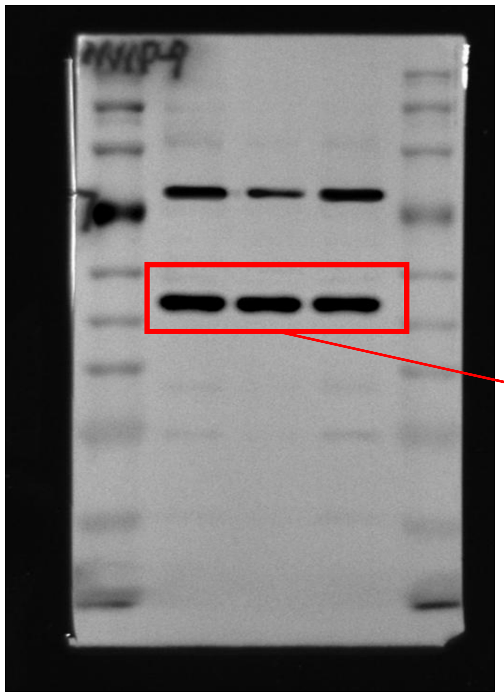

MMP9

When determining the relative expression, each group used its own internal reference protein. The internal reference proteins placed in the figure were randomly selected from the two groups.

Figure 12 C

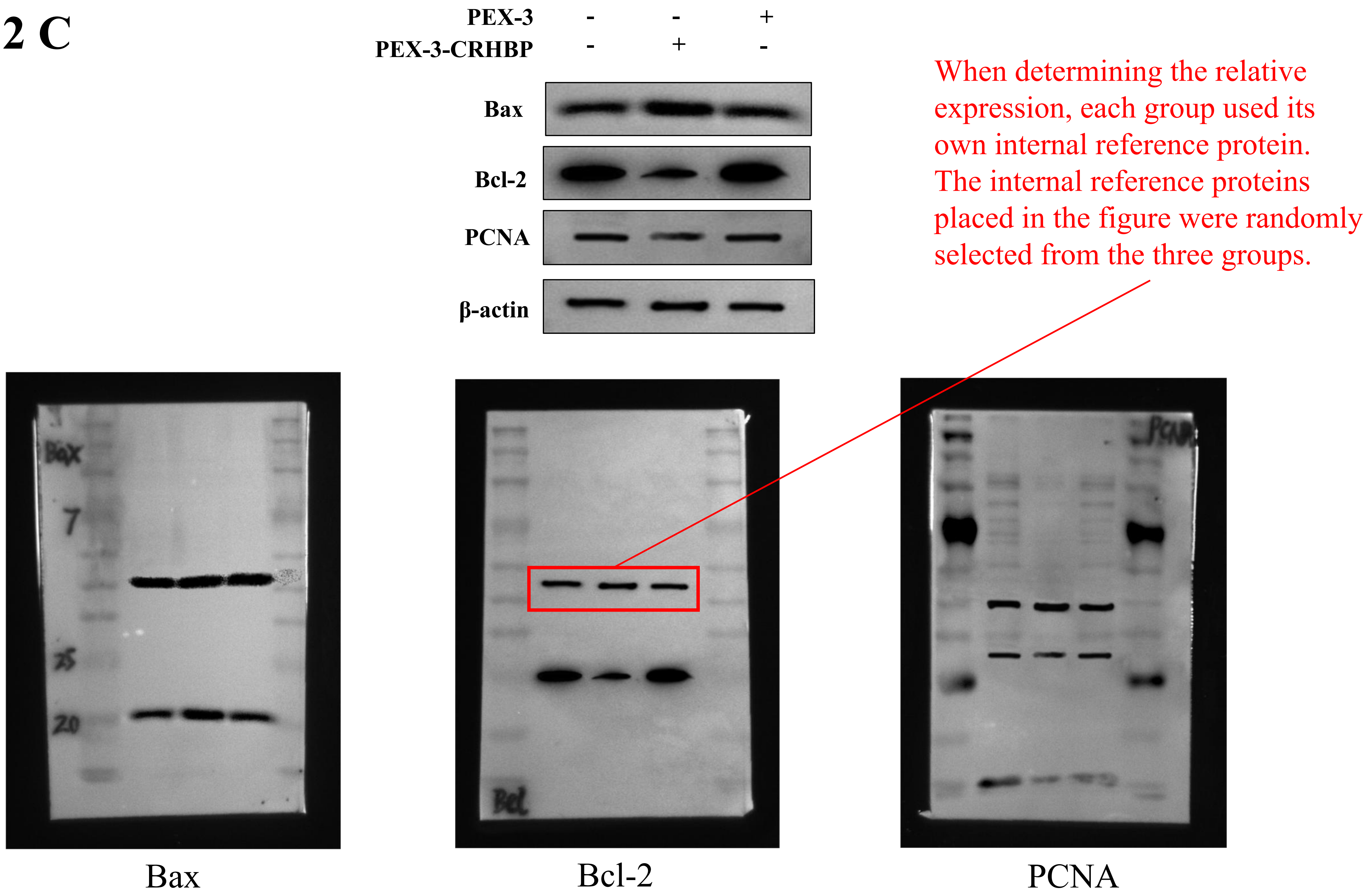
PEX-3 - - +
PEX-3-CRHBP - + -
Bax
Bcl-2
PCNA
β-actin
When determining the relative expression, each group used its own internal reference protein. The internal reference proteins placed in the figure were randomly selected from the three groups.
Bax
Bcl-2
PCNA
